# Supplementary material for: A modular cloning toolkit for genome editing in plants
Source: BMC Plant Biol. 2020 Apr 23;20:179. doi: 10.1186/s12870-020-02388-2 (PMC7178738; doi:10.1186/s12870-020-02388-2)
Supplement: Supplementary file 4 — Additional file 4: Figure S2. A flowchart diagram illustrating assembly of level 2 constructs with gRNAs expressed under individual Pol III promoters. Figure S3. A flowchart diagram illustrating assembly of level 2 constructs with gRNAs expressed as tRNA-sgRNA polycistronic units. Figure S4. Sanger sequencing reads illustrating gene deletions induced by CRISPR/Cas in wheat protoplasts. Figure S5. The unprocessed image of the DNA gel presented in Fig. 6b. [file 12870_2020_2388_MOESM4_ESM.pptx]

## Slide 1
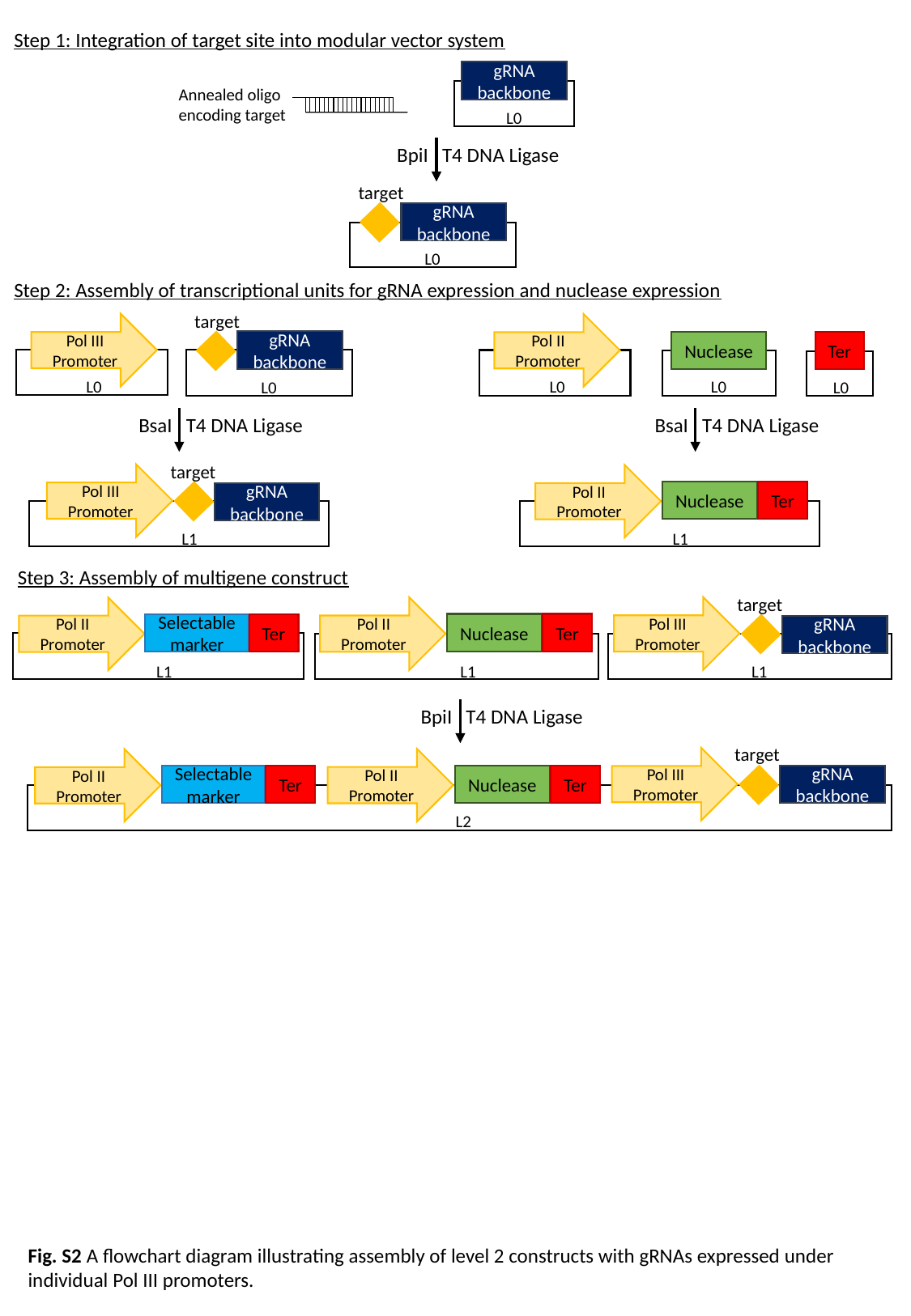

Step 1: Integration of target site into modular vector system
gRNA backbone
Annealed oligo
encoding target
L0
BpiI T4 DNA Ligase
target
gRNA backbone
L0
Step 2: Assembly of transcriptional units for gRNA expression and nuclease expression
target
Pol III Promoter
Pol II Promoter
gRNA backbone
Nuclease
Ter
L0
L0
L0
L0
L0
BsaI T4 DNA Ligase
BsaI T4 DNA Ligase
target
Pol III Promoter
Pol II Promoter
Nuclease
Ter
gRNA backbone
L1
L1
Step 3: Assembly of multigene construct
target
Pol III Promoter
Pol II Promoter
Pol II Promoter
Nuclease
Ter
Selectable marker
Ter
gRNA backbone
L1
L1
L1
BpiI T4 DNA Ligase
target
Pol III Promoter
Pol II Promoter
Pol II Promoter
gRNA backbone
Nuclease
Ter
Selectable marker
Ter
L2
Fig. S2 A flowchart diagram illustrating assembly of level 2 constructs with gRNAs expressed under
individual Pol III promoters.

## Slide 2
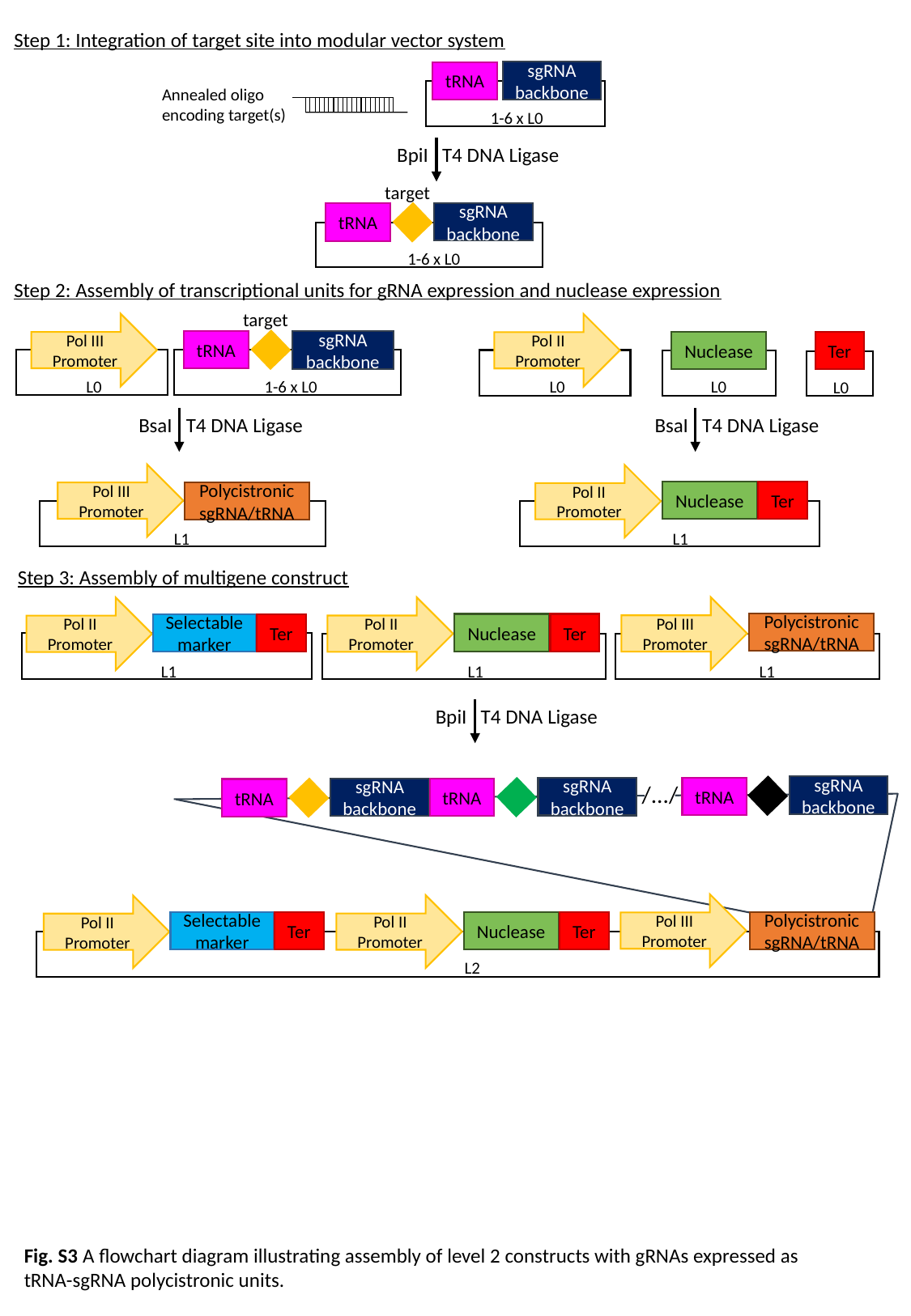

Step 1: Integration of target site into modular vector system
sgRNA backbone
tRNA
Annealed oligo
encoding target(s)
1-6 x L0
BpiI T4 DNA Ligase
target
sgRNA backbone
tRNA
1-6 x L0
Step 2: Assembly of transcriptional units for gRNA expression and nuclease expression
target
Pol III Promoter
Pol II Promoter
tRNA
sgRNA backbone
Nuclease
Ter
L0
1-6 x L0
L0
L0
L0
BsaI T4 DNA Ligase
BsaI T4 DNA Ligase
Pol III Promoter
Pol II Promoter
Nuclease
Ter
Polycistronic sgRNA/tRNA
L1
L1
Step 3: Assembly of multigene construct
Pol III Promoter
Pol II Promoter
Pol II Promoter
Polycistronic sgRNA/tRNA
Nuclease
Ter
Selectable marker
Ter
L1
L1
L1
BpiI T4 DNA Ligase
/.../
sgRNA backbone
tRNA
sgRNA backbone
tRNA
sgRNA backbone
tRNA
Pol III Promoter
Pol II Promoter
Pol II Promoter
Nuclease
Ter
Polycistronic sgRNA/tRNA
Selectable marker
Ter
L2
Fig. S3 A flowchart diagram illustrating assembly of level 2 constructs with gRNAs expressed as
tRNA-sgRNA polycistronic units.

## Slide 3
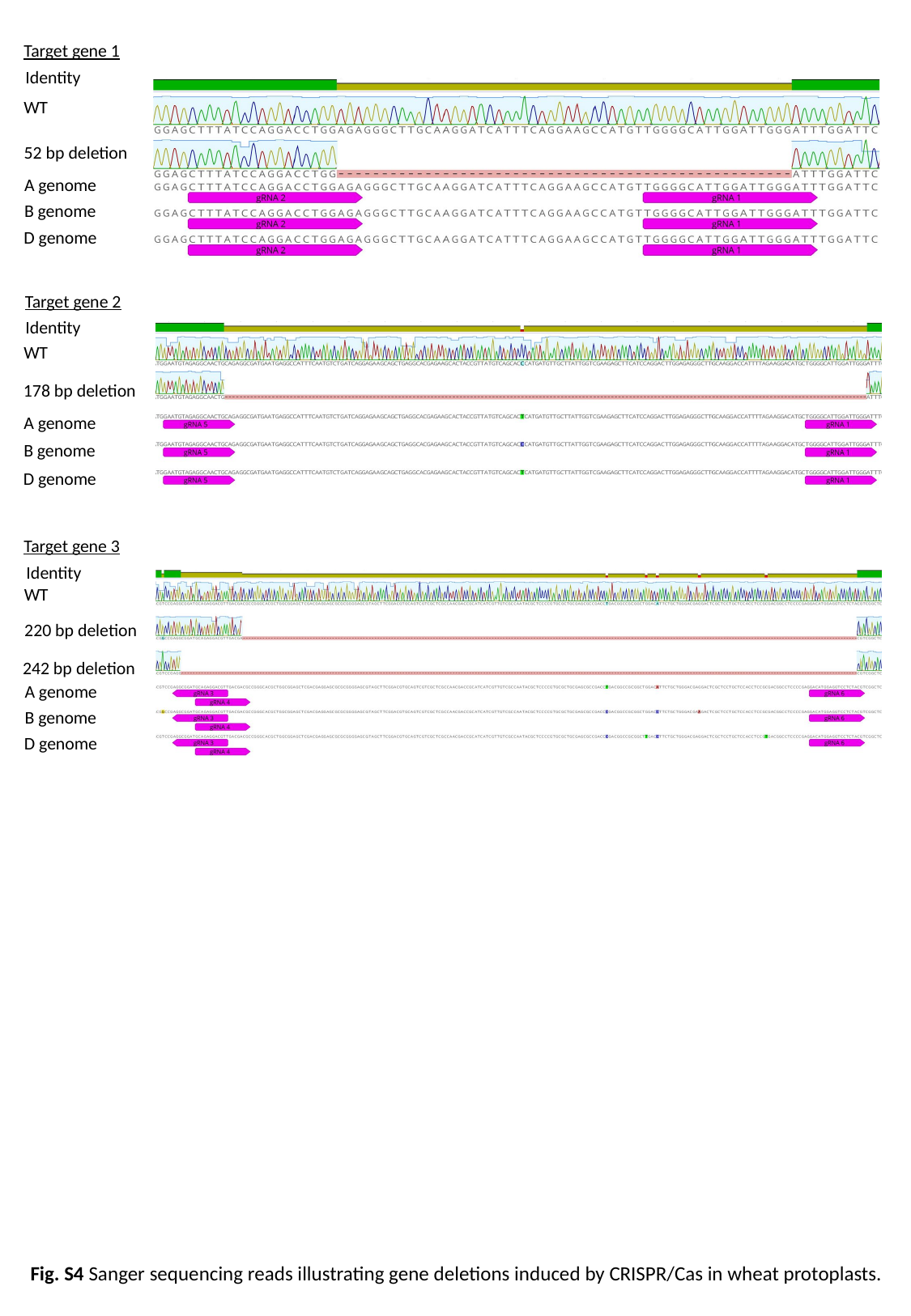

Target gene 1
Identity
WT
52 bp deletion
A genome
B genome
D genome
Target gene 2
Identity
WT
178 bp deletion
A genome
B genome
D genome
Target gene 3
Identity
WT
220 bp deletion
242 bp deletion
A genome
B genome
D genome
Fig. S4 Sanger sequencing reads illustrating gene deletions induced by CRISPR/Cas in wheat protoplasts.

## Slide 4
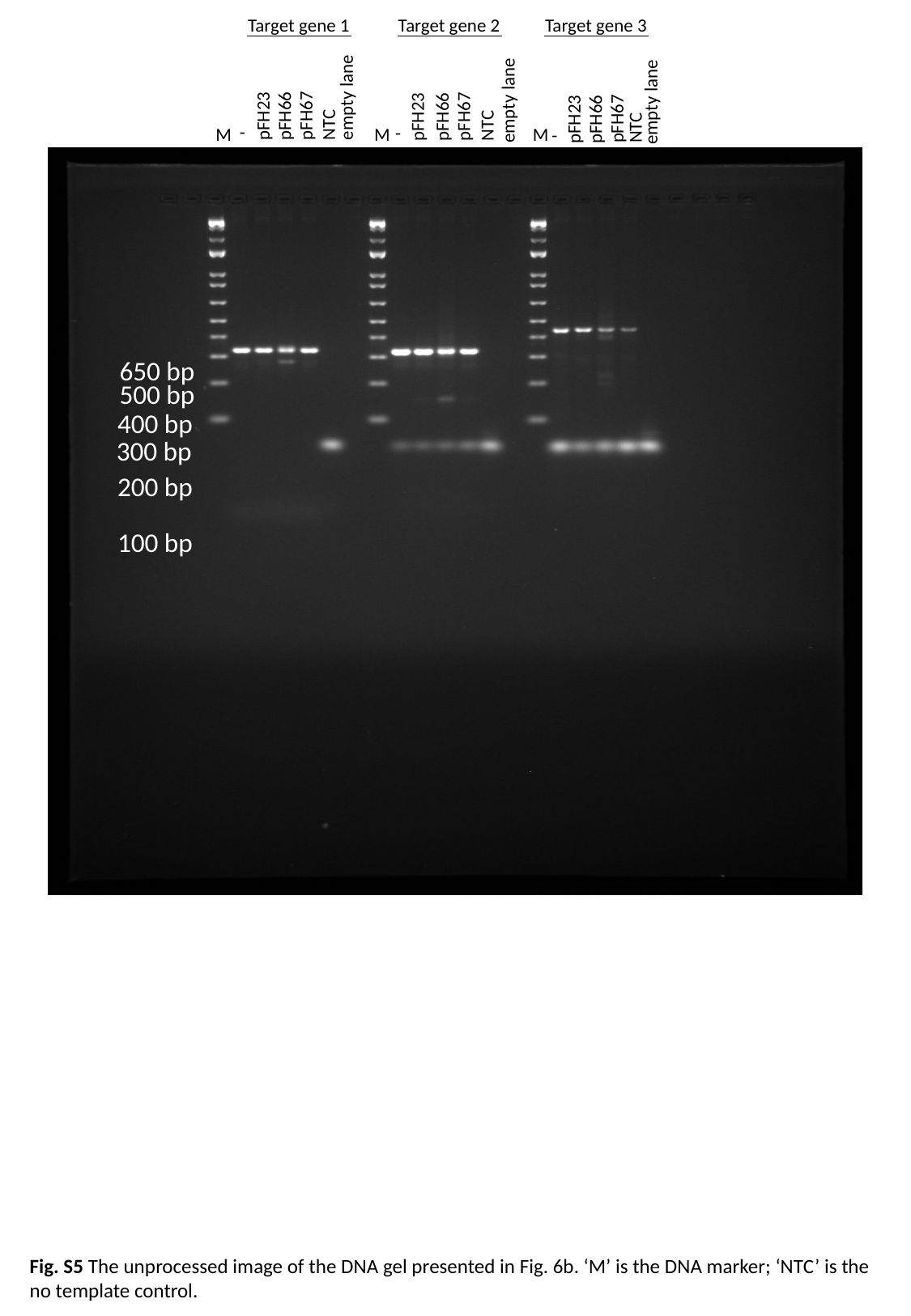

Target gene 1
Target gene 2
Target gene 3
empty lane
empty lane
empty lane
pFH67
NTC
pFH23
pFH66
pFH67
NTC
pFH23
pFH66
pFH67
NTC
pFH23
pFH66
-
-
M
M
M
-
650 bp
500 bp
400 bp
300 bp
200 bp
100 bp
Fig. S5 The unprocessed image of the DNA gel presented in Fig. 6b. ‘M’ is the DNA marker; ‘NTC’ is the
no template control.
